# Health Centres 75+ as a New Model to Improve Care for Older People in Poland

**DOI:** 10.3390/ijerph19127487

**Published:** 2022-06-18

**Authors:** Anna Sagan, Małgorzata Gałązka-Sobotka, Piotr Czauderna, Aldona Frączkiewicz-Wronka, Katarzyna Badora-Musiał, Natalia Petka, Iwona Kowalska-Bobko

**Affiliations:** 1 European Observatory on Health Systems and Policies, London School of Economics and Political Science, London WC2A 2AE, UK; 2 European Observatory on Health Systems and Policies, London School of Hygiene & Tropical Medicine, London WC1E 7HT, UK; 3 Institute of Healthcare Management, Faculty of Economics and Management, Lazarski University, 02-662 Warsaw, Poland; m.galazka-sobotka@lazarski.edu.pl; 4 Faculty of Medicine, Medical University of Gdańsk, 80-210 Gdansk, Poland; piotr.czauderna@gumed.edu.pl; 5 Department of Public Management and Social Sciences, University of Economics in Katowice, 40-287 Katowice, Poland; aldona.fraczkiewicz-wronka@uekat.pl; 6 Institute of Public Health, Faculty of Health Sciences, Jagiellonian University, 31-067 Krakow, Poland; kasia.badora@uj.edu.pl (K.B.-M.); natalia.petka@student.uj.edu.pl (N.P.); iw.kowalska@uj.edu.pl (I.K.-B.)

**Keywords:** geriatric care, healthcare, coordination, integrated care, Poland

## Abstract

According to a recent national audit, the cost of treating patients in geriatric wards is 20–30% less compared to those treated in internal medicine wards. Yet, geriatric care remains largely underdeveloped in Poland, with few human, material, and financial resources. Despite numerous attempts to raise the profile of geriatrics over the years, little progress has been achieved. In 2019, experts under the President of Poland proposed the creation of a network of Health Centres 75+ as the first pillar of geriatric care. These are meant to provide ambulatory services for older people and coordinate provision of other health and social care services at the county level. The goal is to create a community model of care, whereby older people would receive needed services close to their place of residence, allowing them to live independently for as long as possible. Although the proposal has been welcomed by the geriatric community and the patients, the acute shortages of human, physical, and financial resources raise concerns about its feasibility. However, the new strategic plans for the health system propose solutions that appear to be supportive of the new proposal, and the Office of the President is discussing joining forces with the Ministry of Health to improve its chances of implementation. Given the increasing pace of population ageing and underdeveloped provision of geriatric services, these efforts are very much needed.

## 1. Introduction

Poland is still a relatively young country compared to other countries in the European Union (EU). Its per capita gross domestic product (GDP) amounts to less than 80% of the average GDP in the EU. In 2019, the share of people aged 65+ in Poland accounted for 18.2% of total population, which is slightly below the EU average of 20.6% [1]. However, this share has been increasing faster in Poland than in other countries (a 4.6 percentage point increase between 2010 and 2020 compared to a 3 percentage point rise in the EU [2]); and is forecast to reach 30% by 2050, surpassing the projected EU average of 29.6% [3]. People aged 75+ accounted for 7% of the total population in Poland in 2020, compared to 9% in the EU, and these shares are forecast to increase to 51% and 57%, respectively, by 2050 [4]. Since older people often suffer from multiple comorbidities, frailty, and functional limitations to daily living tasks, they require more and substantially different health and care services than younger people [5,6,7]. Geriatric patients in Poland suffer from the following problems, comprising both age-related chronic diseases and geriatric syndromes that are common clinical presentations, such as falls and incontinence, that do not fit into specific disease categories but have substantial implications for functionality and life satisfaction [8]: hypertension (60% of geriatric patients), depression (52%), urinary incontinence (48%), falls (41%), dementia (35%), diabetes (31%), heart failure (27%), peptic ulcer disease (22%), protein and energy malnutrition (20%), delirium (19%), iatrogenic syndromes (17%), chronic kidney disease (17%), Parkinson’s disease (16%), and cancer (95) [9]. Older Poles declare having poor health more often compared to their peers in other countries in Europe—in 2019, 30.8% of Poles aged 65+ reported having bad or very bad health compared to 17.8% in the EU on average [10]. This share increases to 41.8% in people over 75 years of age, compared to 23.8% in the EU. The burden of chronic diseases and multimorbidity is also high among older Poles. According to the data collected within the PolSenior survey, up to 80% of people aged 65+ suffer from more than one condition [11] and people over 70 years old suffer from at least three chronic diseases [12]. In terms of performing activities of daily living, 34.1% of Poles aged 65+ report some or severe limitations compared to 26.1% in the EU (2014 data) [13]. These shares increase to, respectively, 36.6% and 28.8% for people aged 75+. All these factors increase the likelihood of the need for medical services among older people, including nursing and social care services.

Geriatric care offers a holistic approach to multiple health problems of older age that accounts for risks such as adverse consequences of polypharmacy, frailty, and mobility limitations [14,15]. Assessment by specialist geriatric teams, coupled with post-discharge interventions at home, has been linked to shorter hospitals stays, fewer readmissions, and fewer nursing home placements, and there is now considerable evidence that social care interventions, including early transfer to nursing homes or own homes with support from community-based health and social care services, can reduce hospital admission and length of stay [16].

The purpose of this Perspective piece is to describe the latest key policy proposal to improve provision of geriatric care in Poland put forward in 2019, before the outbreak of the COVID-19 pandemic, which focuses on the introduction of a network of ambulatory centres for people aged 75+ as the main pillar of geriatric care. Section 2 provides the policy background and describes earlier efforts to improve care for older people in Poland. Section 3 describes the content of the new policy proposal in more detail. Section 4 discusses factors that may affect its implementation, including by assessing the positions of key stakeholders towards the proposal, should the works on the proposal be resumed if the pandemic is able to be better controlled. Finally, Section 5 concludes and offers policy recommendations.

## 2. Policy Background

**Figure A1:**
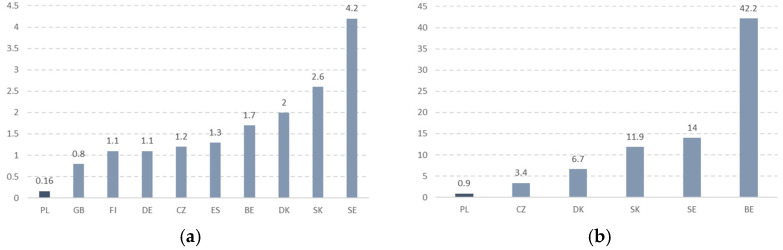
Geriatric healthcare resources in selected European countries, 2009. (**a**) Geriatric specialists per 10,000 people aged 65+. (**b**) Geriatric beds per 10,000 people aged 65+. Source: [40].

Since the early 2000s, numerous calls have been made to develop provision of geriatric care in Poland, with health policy analysts noting the large gap between geriatric resources in Poland compared to some countries in Europe, including the neighbouring Czechia and Slovakia (Figure A1 in Appendix A). Experts from the Polish Society of Gerontology have argued that development of geriatric care is desirable not only for social and ethical, but also for economic reasons, as it can extend the number of years lived in good health and improve functional mobility, thus reducing the need for health and care services [17]. A report by the Supreme Audit Office from 2015 found that the cost of treatment of patients in geriatric wards was 20–30% less than that of patients treated in internal medicine wards, and the annual cost of their pharmaceutical care was 10% lower [18]. Cost-effectiveness of simple geriatric interventions, such as comprehensive geriatric assessment (CGA) or preventive home visits are also well established (e.g., [19,20]).

Yet, geriatric resources have seen little improvement. According to the Supreme Chamber of Physicians and Dentists, in 2019, Poland had 502 physicians with a geriatric specialisation, of whom 488 (or approx. 0.7 per 10,000 people aged 65+) were professionally active [21] (the Ministry of Health provides a slightly lower number—462; [22]). However, it must be noted that only about half of physicians with a specialisation in geriatrics work as geriatricians [23] and that geriatrics is typically the second specialisation chosen by internal medicine or family medicine physicians, whose numbers have been falling over the years [22]. According to the National Consultant in Geriatrics, the recommended number of geriatricians should be 7.8 per 100,000 inhabitants, compared to the current number of 1.2 per 100,000 [22]. The number of nurses specialising in geriatrics in 2019 amounted to 2552 (or approx. 3.7 per 10,000 people aged 65+) [21].

**Figure A2:**
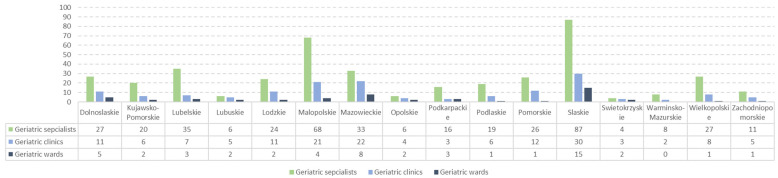
Geriatric healthcare resources in Poland by region, 2019. Note: The number of geriatric specialists shows professionally active geriatricians at the end of 2017 [23]. Source: [41].

Since 2015, there has been at least one geriatric care ward in all regions except one (Warmińsko-mazurskie in the north-east). Most of them are located in the Silesian region (southern Poland), which is forecast to become one of the oldest regions in Poland by 2030 and where geriatrics has been declared a regional priority (Figure A2 in Appendix A) [24]. The Mazowieckie region, where Poland’s capital (Warsaw) is located, had the second highest number of geriatric wards in 2019. Despite these improvements, resources remain insufficient [25]. For example, geriatric clinics can be found in only 41 of 374 counties, with the highest numbers located in Cracow (8) and Warsaw (9) [26].

Numerous efforts have been made over the past two decades to raise the profile of geriatrics in Poland, starting with the recognition of geriatrics as a priority area for specialist medical training (Table 1). These attempts have been led by various actors, including the community of geriatric specialists, the Ministry of Health, the Civil Rights Ombudsman, and the President of Poland, and should be seen in the contexts of the provision of long-term care (LTC) and social care services, which are also seen as largely underdeveloped in Poland [27,28]. The need to strengthen provision of geriatric care has been recognised in numerous conferences, seminars, and reports. For example, a report published in 2015 by the National Audit Office drew attention to the deficits of geriatric services in Poland and the urgent need to build an effective system of care for older people, complete with a support system for their next of kin [18]. These calls were reiterated in another National Audit Office report published in 2021 [9].

Despite these efforts and calls, little actual progress has been made in strengthening the role of geriatrics in the health system. On the contrary, its position has been recently weakened. For example, geriatrics has been omitted from the health need maps developed since 2015, which is a planning tool that is meant to improve contracting of health services by the public payer (the National Health Fund, NHF). Geriatrics is also largely missing from the hospital network introduced in 2017, on the basis of which the NHF contracts hospital services, where geriatrics was constrained to the 3rd level of hospital provision (i.e., mainly regional hospitals). Geriatric wards operating in the 1st and 2nd reference level hospitals (i.e., mainly in county hospitals) are not included in the network, which means they do not benefit from lump-sum contracting that is awarded to hospitals included within the network [29]. Public coverage of geriatric syndromes remains minimal and the few services that are included are underpriced, despite the recognised potential savings that provision of such services could bring [18]. For example, CGA, which is an important element of geriatric care, is not financed at the level of primary and outpatient specialist care, and geriatric rehabilitation is not financed by the NHF (it can be provided at medical nursing homes (Zakład Opiekuńczo–Leczniczy, ZOL) and is financed from the state or paid for privately by the patients).

Given the existing shortages of dedicated geriatric services, older patients usually seek medical care from primary health care (PHC) doctors, constituting the key users of primary care services [18]. Since this patient group often suffers from complex health problems, PHC doctors are compensated with higher capitation rates for older patients on their lists—2.7 times higher for people aged 66–75 and 3.1 times higher for people over 75 years of age. Higher capitation rates are also applied for residents of social welfare homes (Dom pomocy społecznej, DPS) (3.1 times higher) and patients with chronic conditions such as diabetes, cardiovascular diseases, and thyroid diseases (3.2 times higher) [30]. Coordination of health and care services for older patients, and exchange of information between the various elements of the health and social care systems, are perceived to be among the biggest problems faced by the PHC doctors [11].

## 3. Policy Content

**Figure 1:**
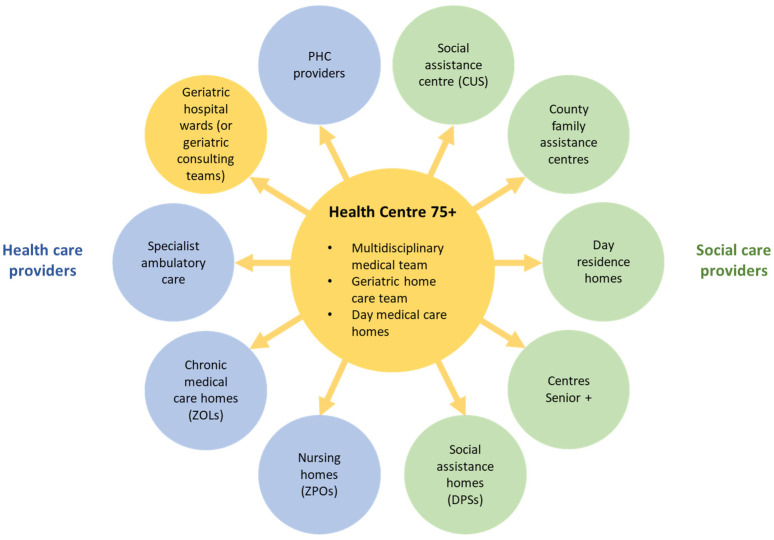
Proposed organisation of care for older people organised around Health Centres 75+. Source: Authors based on [31]. Notes: CUS = Centrum Usług Społecznych (social assistance centre); DPS = dom pomocy społecznej (soccial assistance home); ZPO = zakład pielęgnacyjno-opiekuńczy (nursing home).

In 2019, a team of experts associated with the National Development Council under the President of Poland and the Ministry of Health put forward a new proposal to improve geriatrics and care for older people more generally [31]. Its central idea is the creation of a network of Health Centres 75+—one in each county, i.e., one per about 100,000 inhabitants—providing ambulatory services for older people as the first pillar of geriatric care in Poland. These Centres could operate either out of county hospitals or larger PHC practices. The role of these Centres is to coordinate, in cooperation with family physicians and social assistance institutions, care for older people at the county level (Figure 1). This is meant to include not only geriatric diagnostic and treatment services, but also social care and rehabilitation services, and relevant services provided by the local self-governments and nongovernmental organisations. Older people attending these Centres would be looked after by an interdisciplinary team, which should include, among others, a geriatrician, a psychiatrist, and a physiotherapist, and would be assigned a treatment coordinator. The new Centres will comprise day medical care homes providing medical services for older people requiring rehabilitation services after hospital discharge and home care teams supporting patients at their homes. The model also foresees that the Health Centres 75+ would employ health educators to provide family and other informal carers with basic information on care and medical procedures. The goal is to create a community model of care, whereby medical and social services for older people are provided close to their place of residence, in order to support them in living independently for as long as possible, and to reduce the number of hospitalisations.

Geriatric hospital wards are meant to constitute the second pillar of the new model. The proposal assumes increasing the number of geriatric wards to 100–120, i.e., approximately one ward per 300,000–350,000 inhabitants. This is not meant to lead to an increase in the number of hospital beds; instead, the proposal suggests transformation of some of the existing hospital beds into geriatric care beds. Every geriatric ward is meant to serve 3–4 Health Centres 75+. These wards would take over geriatric patients from other hospital wards, and serve geriatric patients referred to by the Health Centres 75+. After discharge, patient files would be passed to the Centres with recommendations for any follow-up medical care.

The model further assumes that every person aged 75+ would undergo a basic geriatric assessment at the PHC level to detect any significant health problems and determine the degree of independence so that they can start treatment and be connected with other (social care) services they need as early as possible. Patients identified as geriatric patients would undergo a CGA by a geriatric medical team at the Centre led by a treatment coordinator.

## 4. Discussion

The draft law on Health Centres 75+ was expected to be referred to the parliament in the first half of 2020, but this was postponed due to the outbreak of the COVID-19 pandemic. This prevented a broader public debate over the proposal. Nevertheless, positions of the key stakeholders can be discerned from various reports, press articles, and expert opinions.

The Office of the President has clearly been the driving force behind the policy proposal, but needs support from other stakeholders, particularly from the Ministry of Health, to pass the proposal into the law. The proposal has been welcomed by geriatric physicians and patients (as represented, for example, by Patients’ Rights Ombudsman and the Coalition ‘To help the dependent’), but these and other stakeholders share much scepticism about its feasibility [11]. The key reasons for concern are the acute shortages of human and physical resources (geriatricians, geriatric nurses, and geriatric wards) and the very low level of financing of both geriatrics, and long-term (and social) care and rehabilitation services, which are all in in dire need of investment [12]. There is also a lack of clarity about patient pathways at the intersection of the new model with PHC, outpatient specialist care, and social care services; for example, many residents of social welfare homes are currently not registered with a family doctor [32,33].

The position of the PHC physicians and the counties has so far been ambivalent since there is no detailed information about the patient pathways within the new model or about its funding. PHC doctors may see the Centres as an opportunity to relieve them of some of their duties, while at the same time ensuring better care for geriatric patients and improving coordination of services [11,34]. However, since they also receive high capitation rates for treating older patients (see above), they may be reluctant to lose this stream of income. The counties, who are meant to be the founders of the new Centres, may not be inclined to take on more responsibilities without receiving additional funding, as their health budgets are currently very limited [35,36]. At the same time, they may be under pressure to respond to the demands of their local populations, which are increasingly older and progressively better organised, such as in the Senior Councils, which have increased in numbers in recent years, and which can exert influence over social and health policies [37,38].

The Ministry of Health, the stakeholder with the most influence in the system, has so far not shown much support for the project. In addition to the reasons outlined above, other reasons for this may include poor cooperation between the Ministry and the social care sector, and the lack of clarity about the division of costs between health and social care in the proposed model. There is also a concern that the introduction of a dedicated solution for geriatric patients may lead to demands for similar solutions from other population groups. Finally, the Ministry may not feel much ownership over the project since it was driven by the Office of the President.

The National Reconstruction Plan, which at the time of writing (May 2022) was yet to be approved by the European Commission, will—if approved—trigger a release of funds from the EU’s Reconstruction Fund that can be used for implementing health sector and other reforms. These are set out in the National Transformation Plan for 2022–2026 published in late 2021, which is an executive act and has been guided by the framework document for the health sector—’Healthy future. Strategic framework for the development of the health care system for the years 2021–2027, with a perspective until 2030’. Although the Plan does not directly support the creation of Health Centres 75+, it makes a series of recommendations in the area of geriatrics and long-term care that are compatible with this policy idea. With respect to geriatrics, the Plan focuses on improving quality of and access to hospital care, calling for a transformation of at least 850 hospital beds in wards with low occupancy rates into geriatric beds. With respect to long-term care, the Plan foresees several actions including: (1) transformation of some of existing hospital beds into long-term care beds providing residential nursing and care services; (2) development of community long-term care in Day Medical Care Homes (Dzienny Dom Opieki Medycznej, DDOM) and inclusion of services provided in these homes in the basket of guaranteed services; (3) inclusion of long-term care services provided by medical caregivers (Table 1) in the basket of guaranteed services; and (4) development of a training programme and a psychological support programme for informal carers who look after older people with limitations in daily living activities. All these measures can be seen as supportive from the perspective of the proposed Health Centres 75+ model.

The Plan also foresees a range of measures to strengthen PHC, including by improving coordination of care, home care, and health promotion. These activities will be informed by learning from the pilot of the PHC PLUS model, which ended in late 2021 (Table 1). This can also be regarded as a positive development, given that the originators of the Health Centre 75+ proposal see PHC as a bridge that that can support development of the Centres before they become fully functional [11]: since family doctors play the role of gatekeepers in the Polish health care system, directing patients to the needed specialist curative and other services, they can, in a similar fashion, direct older patients (e.g., with the use of special screening tests) to services provided at the Health Centres 75+. This could be supported by appropriate adaptations of the specialisation training in family medicine and is seen as a potential means to counter the existing shortages of geriatric staff [11].

The pilot of the Mental Health Centres, which has been implemented since mid-2018 [39], can be seen as an inspiration for the implementation of Health Centres 75+: both geriatric and mental health care suffer from similar problems (relatively low numbers of health professionals, low financing) and require much investment; both Health Centres 75+ and Mental Health Centres involve cooperation with PHC, specialist health services, and social care—the former could thus learn from the experiences of the latter.

Although the initial lack of support from the Ministry of Health has brought the policy proposal to an impasse, the Office of the President and the Ministry of Health are now considering joining forces to overcome it and to rework the policy idea into a joint proposal. This may give it a real chance of being implemented and provide a major opportunity for the development of comprehensive care for older people in Poland.

## 5. Conclusions

Geriatric care in Poland has found itself in a vicious circle for many years: it has suffered from chronic shortages of geriatric beds and specialists but, at the same time, the numbers of training places and job vacancies have been very low, discouraging medical students from entering the profession and not motivating further investment in geriatric infrastructure. Although the outbreak of the COVID-19 pandemic meant that work on the draft law on Health Centres 75+ was suspended, the pandemic has drawn more attention to the health and social needs of older people in Poland, offering a window of opportunity to resume the debate about improving provision of services for this population group. The proposed draft seeks to create a community model of care for older people to allow them to live independently for as long as possible and receive the comprehensive, coordinated services they need. Although the proposal has some drawbacks, population ageing and the current lack of geriatric resources make it an important initiative that is worth putting back on the policy agenda and giving it a prominent place in the post-pandemic recovery plans.

## Figures and Tables

**Table 1:** Key policy changes in geriatric care and related areas, 2000–2021.

Year	Area	Policy Content
2003	Geriatrics	Since 2003, geriatrics has been recognised as a priority medical specialisation, which means that providers offering residency places in geriatrics are eligible for a monthly financial supplement
2007	Geriatrics	A working group was established by the Ministry of Health to prepare geriatric standards and proposals for system-level changes in geriatric care; these have been developed but not implemented
2007	Long-term care	The profession of medical caregiver was introduced to provide nursing and care services to ill and dependent persons
2008	Geriatrics	The Ministry of Health hosted a conference on the development of geriatrics in Poland
2009	Long-term care	A proposal to create a system of mandatory LTC insurance was considered by the Senate (the upper house of the Polish Parliament); the proposal was abandoned due to financial concerns
2010	Geriatrics	The Ministry of Health prepared a draft ordinance on the standards of conduct in the provision of health services in the field of geriatrics; the draft was not signed
2011	Long-term care	A proposal to establish nursing vouchers for LTC was considered by the Senate; the proposal was abandoned due to financial concerns
2012	Monitoring/needs assessment	Publication of the first edition of PolSenior study, analysing the situation of older people in Poland
2012	Social activation of older people	Start of the Government Programme for the Social Activation of Older People (ASOS) for 2012–2013
2012	Social activation of older people	Adoption of the ‘Pact for Seniors’ by the National Congress of the Universities of the Third Age
2013	Social activation of older people	Amendment of the act on the municipal government regulates establishment of municipal Seniors Councils
2013	Social policy/Social activation of older people	Adoption of the ‘Foundations for the Long-term Seniors Policy’ for 2014–2020; Adoption of ‘Generational Solidarity–Increasing Economic Activity for People aged 50+’ programme; Start of the Government Programme for the Social Activation of Older People (ASOS) for 2014–2020
2013	Geriatrics	Polish Geriatric Society developed standards of practice in geriatric care; standards were not implemented
2013	Social policy	Establishment of the Senior Policy Council as a consultative and advisory body for the Ministry of the Family and Social Policy
2014	Social policy	Establishment of the Parliamentary Commission on Senior Policy focusing on the monitoring of the living conditions and social services for older people
2015	Geriatrics	A report on the medical care for older people was published by the National Audit Office
2015	Geriatrics	A fast-track specialisation training in geriatrics was established
2015	Geriatrics	Establishment of the National Institute of Geriatrics, Rheumatology, and Rehabilitation
2015	Social policy	Inauguration of the Civic Seniors’ Parliament during a plenary session of the lower house of the Polish Parliament
2015	Monitoring/needs assessment	Health needs maps were introduced as a planning tool to improve contracting of services in the regions (which were tasked with their preparation); geriatrics was not included in these maps
2015	Social activation of older people	Programme ‘Senior+’ for 2015–2020 was introduced by the Ministry of the Family and Social Policy, offering financial support for the local self-governments to support investments in promoting active social life by expanding local support centres such as day homes and clubs for people aged 60+ (the programme has been extended for the 2021–2025 period; see below)
2015	Monitoring/needs assessment	The Act on Older Persons prepared by the Parliamentary Commission on Senior Policy defined the scope of monitoring of the situation of older people in Poland
2016	Geriatrics	The Civil Rights Ombudsman hosted a seminar on the condition of geriatrics in Poland
2016	Long-term care	Public financing for long-term day care services in day medical care homes (known as DDOM in Polish) was introduced
2017	Hospital care	Hospital network was introduced to rationalise provision of hospital services; geriatrics was not included in hospitals qualified for the 1st and 2nd level of hospital provision (i.e., mostly county and some city hospitals)
2017	Access to medicines	People aged 75+ were granted free access to a broad range of medicines
2017	Care coordination	The Ministry of Health in cooperation with the World Bank developed several models to improve coordination of care, including one focusing on PHC (see below) and one on improving integration between health care and social care services for older people; the latter was not implemented
2017	Primary health care	A new model of PHC provision developed jointly with the World Bank (called PHC PLUS) was piloted between 2017 and 2021 targeting population 20–65 years old; it introduced multidisciplinary care teams, care coordinators and individual disease management plans for patients with 11 most prevalent non-communicable conditions
2018	Geriatrics	The Civil Rights Ombudsman hosted another seminar on the condition of geriatrics in Poland and appointed the Expert Commission on Older People to assess the situation of older people in Poland
2018	Social services	Programme ‘Opieka 75+’ (Care 75+) introduced by the Ministry of the Family and Social Policy offers subsidies to the municipalities to support investments targeted at improving access to care services for people aged 75+ (the programme has so far been renewed in 2019, 2020, and 2021)
2018	Geriatrics	The National Development Council under the President of Poland recommended thar the Ministry of Health takes action to stop the decline of geriatrics in Poland
2019	Disability support	The State Fund for Rehabilitation of Persons with Disabilities (PFRON) introduced two new programmes: Care services for people with disabilities (Usługi opiekuńcze dla osób niepełnosprawnych) and Respite care (Opieka wytchnieniowa)
2019	Disability support	The Ministry of Family and Social Policy introduced two new programmes: ‘Care and residential centres’ (centra opiekuńczo-mieszkalne), which supports establishment of centres providing services to adults with moderate or significant disabilities, and ‘Personal assistant of a disabled person’ (asystent osobisty osoby niepełnosprawnej), which focuses on providing assistance with activities of daily living, including cultural and other social activities, to disabled persons
2019	Disability support	The Social Insurance Institution (ZUS) introduced monthly supplementary benefit (PLN 500) for disabled people who are incapable of living independently
2019	Geriatrics	Experts associated with the National Development Council and the Ministry of Health put forward a policy proposal to introduce Health Centres 75+
2019	Social services	The Act on the Provision of Social Services by a Social Services Centre, which supports coordination and integration of social services provision at the local level by allowing the municipalities to voluntarily establish Social Services Centres, was signed into law
2020	Geriatrics	The Expert Commission on Older People under the Civil Rights Ombudsman published its final report ‘Situation of older persons in Poland—challenges and recommendations’
2020	Social activation of older people	Establishment of a multiannual programme for older people “The Active+” 2021–2025, as a continuation of the ASOS programme
2020	Social activation of older people	Programme ‘Senior+’ was extended for the period 2021–2025
2021	Geriatrics	Another report on the medical care for older people was published by the National Audit Office
2021	Long-term care	The duration of training for medical caregivers was extended from 1 to 1.5 years and training is now offered solely as full time (rather than evening or weekend) studies
2021	Investment planning	The National Transformation Plan for 2022–2026 was published by the Ministry of Health outlining proposals for targeted investments in the health sector, including geriatrics and LTC, with co-financing from the EU
2021	Primary health care	Introduction of care coordinators became mandatory in all PHC practices; to date, no other elements of the PHC PLUS pilot (see above) have been implemented
2021	Monitoring/needs assessment	Publication of the second edition of PolSenior study, analysing the situation of older people in Poland
2021	Social services	The strategy for the development of social services, public policy for the years 2021–2035, supporting the development of local social services for disadvantaged people, including for older people, was published by the Ministry of the Family and Social Policy

## Data Availability

Not applicable.
